# Application of Coenzyme Q10 for Accelerating Soft Tissue Wound Healing after Tooth Extraction in Rats

**DOI:** 10.3390/nu6125756

**Published:** 2014-12-10

**Authors:** Toshiki Yoneda, Takaaki Tomofuji, Yuya Kawabata, Daisuke Ekuni, Tetsuji Azuma, Kota Kataoka, Muneyoshi Kunitomo, Manabu Morita

**Affiliations:** 1 Department of Preventive Dentistry, Okayama University Graduate School of Medicine, Dentistry and Pharmaceutical Sciences, 2-5-1 Shikata-cho, Kita-ku, Okayama 700-8558, Japan; E-Mails: de17057@s.okadai.jp (T.Y.); de18019@s.okayama-u.ac.jp (Y.K.); dekuni7@md.okayama-u.ac.jp (D.E.); tetsuji@md.okayama-u.ac.jp (T.A.); de18017@s.okayama-u.ac.jp (K.K.); de19013@s.okayama-u.ac.jp (M.K.); mmorita@md.okayama-u.ac.jp (M.M.); 2 Advanced Research Center for Oral and Craniofacial Sciences, Okayama University Dental School, 2-5-1 Shikata-cho, Kita-ku, Okayama 700-8558, Japan

**Keywords:** coenzyme Q10, wound healing, inflammation, alveolar socket

## Abstract

Accelerating wound healing after tooth extraction is beneficial in dental treatment. Application of antioxidants, such as reduced coenzyme Q10 (rCoQ10), may promote wound healing after tooth extraction. In this study, we examined the effects of topical application of rCoQ10 on wound healing after tooth extraction in rats. After maxillary first molars were extracted, male Fischer 344 rats (8 weeks old) (*n* = 27) received topical application of ointment containing 5% rCoQ10 (experimental group) or control ointment (control group) to the sockets for 3 or 8 days (*n* = 6–7/group). At 3 days after extraction, the experimental group showed higher collagen density and lower numbers of polymorphonuclear leukocytes in the upper part of socket, as compared to the control group (*p* < 0.05). Gene expression of interleukin-1β, tumor necrosis factor-α and nuclear factor-κB were also lower in the experimental group than in the control group (*p* < 0.05). At 8 days after tooth extraction, there were no significant differences in collagen density, number of polymorphonuclear leukocytes and bone fill between the groups. Our results suggest that topical application of rCoQ10 promotes wound healing in the soft tissue of the alveolar socket, but that rCoQ10 has a limited effect on bone remodeling in rats.

## 1. Introduction

Tooth extraction is a common dental treatment. However, it can lead to adverse effects such as bacteremia and excess inflammation. Furthermore, prolonged wound healing is disadvantageous for subsequent prosthodontic treatment after tooth extraction. Therefore, acceleration of wound healing after tooth extraction has potential benefits for dentists. 

In the wound healing process, production of reactive oxygen species (ROS) is necessary as a defence against bacterial pathogens [1,2]. However, exposure to excessive ROS also induces oxidative stress and impairs wound healing [3]. This indicates that improvement of local oxidative stress accelerates wound healing. In fact, a recent study revealed that the anti-inflammatory and antioxidant potential of curcumin induced more rapid and improved wound healing in diabetic rats [4]. 

Coenzyme Q10 (CoQ10) is a vitamin-like, oil-soluble molecule. Its reduced form (rCoQ10) is an effective fat-soluble antioxidant [5,6] and an essential element of the mitochondrial respiratory chain [7,8]. Therefore, rCoQ10 may have healing effects on wound tissues by decreasing oxidative stress and improved mitochondrial efficiency. A previous study demonstrated that oral administration of CoQ10 induced synthesis of collagen on injured skin tissue, and had positive effects on cutaneous healing in mice [9]. However, the effects of rCoQ10 on wound healing after tooth extraction remain unclear.

In the present study, we hypothesized that rCoQ10 improves inflammation, collagen density and bone fill after tooth extraction. Such conditions may also stimulate tissue regeneration, which is assessed based on expression of fibroblast growth factor-2 (FGF-2) [10,11,12]. The purpose of this study was to investigate the effects of topical application of rCoQ10 on alveolar socket healing after tooth extraction in rats. 

## 2. Experimental Section

### 2.1. Animals and Diets

Forty male Fischer 344 rats (age, 8 weeks) were housed in an air-conditioned room (23–25 °C) with a 12-h light-dark cycle. They had free access to powdered food (MF, Oriental Yeast Co., Ltd., Osaka, Japan) and drinking water. Experimental protocols were approved by the Animal Research Control Committee of Okayama University (OKU-2013410).

### 2.2. Pilot Study

Rats were randomly divided into four groups; control group (*n* = 3), 0.1% experimental group (*n* = 3), 1.0% experimental group (*n* = 3) and 5.0% experimental group (*n* = 4). Under general anesthesia (2%–4% isoflurane delivered in O_2_ gas), ointment containing rCoQ10 was topically applied to periodontal tissue in rats in the experimental groups (rCoQ10 concentrations were 0.1%, 1.0% or 5.0%) (Kaneka Co., Osaka, Japan) [13]. Control groups received ointment without rCoQ10. Ointment was applied with a cotton ball and did not have any medicinal properties. In all groups, applied ointment was wiped off after 10 min. Rats received topical application of ointment for 7 days. After the experimental period, animals were sacrificed under general anesthesia, and maxillary periodontal tissues were collected. Periodontal tissue levels of total CoQ10 (rCoQ10 and oxidized CoQ10) were quantified by high-performance liquid chromatography.

### 2.3. Experimental Design

Rats were randomly divided into four groups; 3-day control group (*n* = 6), 8-day control group (*n* = 7), 3-day experimental group (*n* = 7), and 8-day experimental group (*n* = 7). 

Left and right maxillary first molars were extracted in all rats under general anesthesia (2%–4% isoflurane delivered in O_2_ gas) with a dental explorer and extracting forceps (#76N; World Precision Instruments Inc., Sarasota, FL, USA). After hemostasis using a cotton ball, rats received topical application of ointment containing 5% rCoQ10 (Kaneka Co., Osaka, Japan) or control ointment without rCoQ10 on the outside of extraction sockets. Ointment with or without rCoQ10 was applied with a cotton ball once a day for 3 or 8 days. Ointment included bee wax (33 g per 100 g ointment) and soybean oil (67 g per 100 g ointment). Applied ointment was wiped off using a cotton ball after 10 min. After 3 or 8 days, animals were sacrificed under general anesthesia with diethyl ether.

### 2.4. Histological Analysis

For histological analysis, the right maxillary molar regions were resected *en bloc* from each rat and were fixed in 4% paraformaldehyde in 0.1 mol/L phosphate buffer (pH 7.4) for 1 day. Animals with remaining palatal roots were excluded. After fixation with paraformaldehyde, maxillary samples were decalcified with 10% tetrasodium-EDTA aqueous solution (pH 7.4) for 2 weeks at 4 °C. Formalin-fixed tissue samples were embedded in paraffin following dehydration with ethanol (70%, 80%, 90%, and 100%) and immersion in xylene. Paraffin-embedded longitudinal sections (6 μm) from palatal roots were then stained with hematoxylin and eosin, Mallory’s aniline blue or other stains described below. 

Immunohistochemical staining of FGF-2 and 8-hydroxydeoxyguanosine (8-OHdG) (an indicator of oxidative stress) were performed. A commercial kit (Histofine Simple Stain MAX PO; Nichirei Co., Tokyo, Japan) was used to determine the levels of FGF-2 expression. Polyclonal antibodies against FGF-2 (Santa Cruz Biotechnology, Inc., Santa Cruz, CA, USA) and 8-OHdG (Chemicon International, Temecula, CA, USA) were diluted 1:200 in phosphate-buffered saline [14,15]. Color was developed with 3-3′-diaminobenzidine tetrahydrochloride. Sections were counterstained with Mayer’s hematoxylin. FGF-2 staining was observed at 100× magnification.

**Figure 1:**
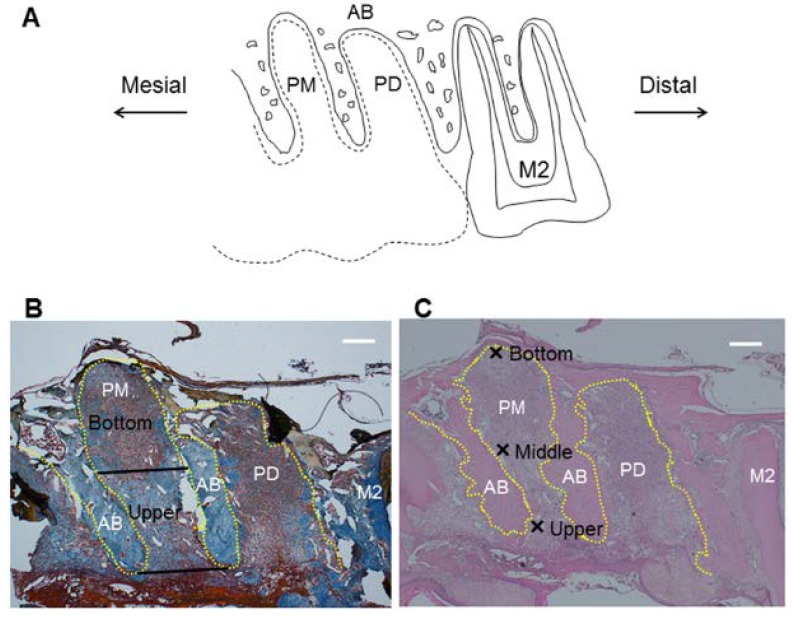
Measurement regions for histological analysis in the socket after tooth extraction. Sagittal plane of region of interest is shown in (**A**)—Black dotted line represents extracted maxillary first molar. Regions to evaluate collagen density or FGF-2 expression are shown in (**B**)—Black straight lines of the alveolar socket represent border of upper area and bottom area. Regions for counting number of polymorphonuclear leukocytes are shown in (**C**)—Crosses represent upper, middle and bottom parts. Scale bar = 400 μm. Yellow dotted line: outline of alveolar socket. AB: alveolar bone; PM: alveolar socket at palatal mesial root of maxillary first molar; PD: alveolar socket at palatal distal root of maxillary first molar; M2: maxillary second molar.

A blinded examiner performed the following histological analysis with a light microscope. Three sections stained with hematoxylin and eosin from each rat were selected for the analyses. Number of polymorphonuclear leukocytes was measured at 400× magnification at three sites within 100 μm of the bone surface in the socket as follows (Figure 1): upper part, alveolar crest; middle part, middle point of cervical-apical direction from alveolar crest to bottom of the socket; and bottom part; bottom of the socket. Each area was 100 μm square. 

Collagen density was determined in tissue sections stained with Mallory’s aniline blue. Three histological sections from each rat were analyzed under a standard microscope equipped with a digital camera at 20× magnification. Images generated by the camera were transferred to a microcomputer and analyzed using mathematical morphology software (WinROOF; Mitani Co., Fukui, Japan) [16]. To analyze collagen density, the socket was divided into two areas, the upper area and bottom area (Figure 1).

### 2.5. Real-Time RT-PCR

Gingival biopsy samples of the left extraction wound were homogenized using a frozen cell crusher (Microtec Co., Chiba, Japan). Total RNA was isolated from the gingival biopsy samples using Trizol reagent (Invitrogen, Carlsbad, CA, USA), in accordance with the manufacturer’s instructions. Isolated RNA was quantified by measuring the absorbance at 260 nm, and purity was determined by the 260/280 nm absorbance ratio. Only samples with a ratio of >1.8 were used [17]. Total RNA was reverse-transcribed by using AMV Reverse Transcriptase (Takara Bio Inc., Shiga, Japan) at 42 °C for 30 min. Real-time PCR was performed by using SYBR Green Real-time PCR Master Mix (Toyobo, Osaka, Japan) in a real-time QPCR system (Agilent Technologies, Tokyo, Japan). Primer sequences of β-actin, matrix metalloproteinase-3 (MMP-3), tissue inhibitor of metalloproteinase-1 (TIMP-1), nuclear factor-κB (NF-κB), interleukin-1β (IL-1β), tumor necrosis factor-α (TNF-α) and heme oxygenase-1 (HO-1) are shown in Table 1.

**Table 1:** Primer sequences used in this study.

Primer	Forward (5′–3′)	Reverse (5′–3′)	Length (bp)	Accession No.
MMP-3	TGGGAAGCCAGTGGAAATG	CCATGCAATGGGTAGGATGAG	81	NM_133523
TIMP-1	CTGAGAAGGGCTACCAGAGC	GTCATCGAGACCCCAAGGTA	88	NM_053819
NF-κB	CACTCTCTTTTTGGAGGT	TGGATATAAGGCTTTACG	206	NM_199267
IL-1β	CACCTCTCAAGCAGAGCACAGA	ACGGGTTCCATGGTGAAGTC	81	NM_031512
TNF-α	TGGGCTCATACCAGGGCTTGAG	CGTCAGCCGATTTGCCATTTC	116	NM_012675
HO-1	GGTGTCCAGGGAAGGCTTTA	GGGGCATAGACTGGGTTCTG	105	NM_012580.2
β-actin	TGTTGCCCTAGACTTCGAGCA	GGACCCAGGAAGGAAGGCT	155	NM_007393

MMP-3: matrix metalloproteinase-3; TIMP-1: tissue inhibitor of metalloproteinase-1; NF-κB: nuclear factor-κB; IL-1β: interleukin-1β; TNF-α: tumor necrosis factor-α; HO-1: heme oxygenase-1.

Amplification conditions were as follows: 45 cycles at 95 °C (30 s), 58 °C (30 s), 72 °C (30 s) for MMP-3; 45 cycles at 95 °C (30 s), 58 °C (30 s), 72 °C (30 s) for TIMP-1; 45 cycles at 95 °C (15 s), 54 °C (20 s), 72 °C (20 s) for IL-1β and NF-κB; 50 cycles at 95 °C (30 s), 55 °C (30 s), 72 °C (10 s) for TNF-α; 50 cycles at 95 °C (30 s), 63 °C (60 s), 72 °C (60 s) for HO-1; and 45 cycles at 95 °C (10 s), 54 °C (20 s), 72 °C (10 s) for β-actin. The mRNA levels were calculated in terms of relative copy number ratio of each mRNA to β-actin for each sample.

### 2.6. Micro Computed Tomography Assessment of Maxillae

Left maxillae from 8-day rats were scanned (TDM1000; Yamato Scientific Co., Ltd., Tokyo, Japan). Computed tomography was performed with the following settings: voltage, 90 kV; electrical current, 20 μA; slice thickness, 8.598 μm. After scanning, three-dimensional (3D) models of the maxillae were prepared using a 3D image-analysis system (TRI/3D-BON64; Ratoc System Engineering, Tokyo, Japan) [18].

### 2.7. Statistical Analysis

Results are presented as mean values ± standard deviation (SD). Student’s *t*-test was used for statistical comparisons between control and experimental groups. α-levels of 0.05 were considered to be statistically significant.

## 3. Results

### 3.1. Pilot Study

Periodontal concentrations of total CoQ10 (*n* = 3–4, mean ± SD, μg/g) following rCoQ10 application for 7 days were 3.3 ± 1.2 in the control group, 4.6 ± 1.6 in the 0.1% experimental group, 4.0 ± 0.6 in the 1.0% experimental group, and 5.1 ± 0.4 in the 5.0% experimental group. Total CoQ10 concentrations in the periodontal tissue increased in the 5.0% experimental group. There was a significant difference in total CoQ10 concentration between the control and the 5.0% experimental groups (*p* < 0.05).

### 3.2. Histological Analysis

**Figure 2:**
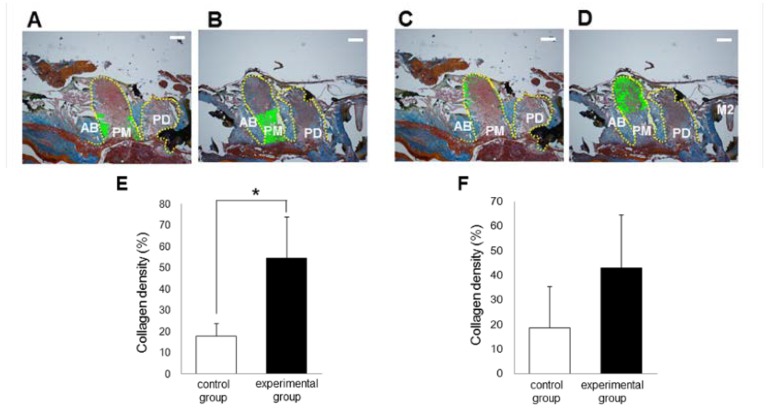
Photographs of alveolar sockets stained with Mallory’s aniline blue in the upper (**A**: control group; **B**: experimental group) and the bottom (**C**: control group; **D**: experimental group) regions at 3 days (Scale bar = 400 μm). In this study, we measured collagen density in the green regions. In the upper region, collagen density was higher in the experimental group than in the control group (**E**); in the bottom region, there were no significant differences in collagen dentistry between the groups (**F**). Bars represent means ± SD of 5–6 rats. * *p* < 0.05 *vs.* control group. Yellow dotted line: outline of alveolar socket. AB: alveolar bone; PM: alveolar socket at palatal mesial root of maxillary first molar; PD: alveolar socket at palatal distal root of maxillary first molar; M2: maxillary second molar.

**Figure 3:**
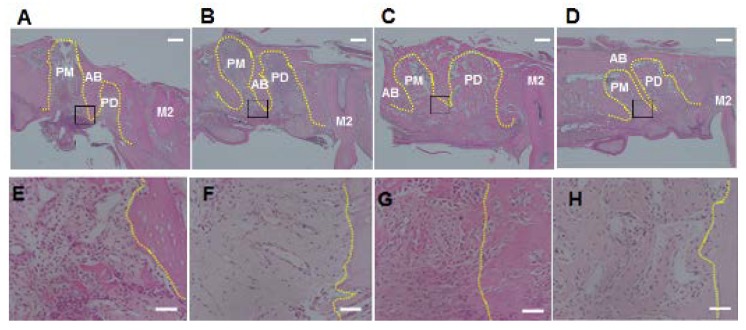
Photographs of alveolar sockets stained with hematoxylin and eosin at 3 days (**A** and **E**: control group; **B** and **F**: experimental group) and 8 days (**C** and **G**: control group; **D** and **H**: experimental group) (Scale bar = 400 μm (**A**–**D**) and 50 μm (**E**–**H**)). The number of polymorphonuclear leukocytes was low in the experimental group compared with the control group at 3 days. On the other hands, both the groups showed low number of polymorphonuclear leukocytes at 8 days. Yellow dotted line: outline of alveolar socket. Black square: areas of **E**–**H**; AB: alveolar bone; PM: alveolar socket at palatal mesial root of maxillary first molar; PD: alveolar socket at palatal distal root of maxillary first molar; M2: maxillary second molar.

**Figure 4:**
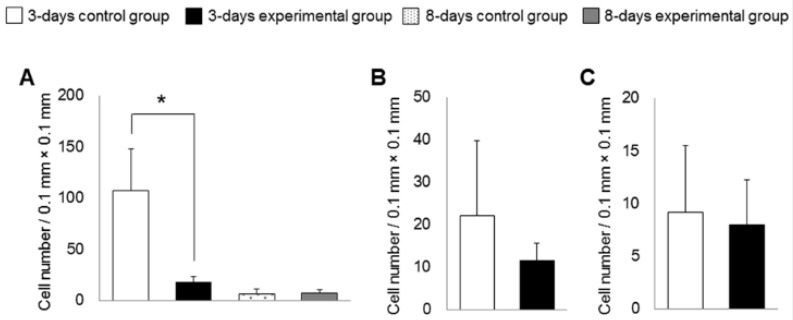
Numbers of polymorphonuclear leukocytes in alveolar sockets. In the upper region (**A**), but not the middle (**B**) and the bottom (**C**) regions, the number of polymorphonuclear leukocytes was lower in the experimental group than in the control group at 3 days. In the upper regions, there were no significant differences in number of polymorphonuclear leukocytes between the groups at 8 days. Bars represent means ± SD of 5–6 rats. * *p* < 0.05 compared with control group.

The results for collagen density and number of polymorphonuclear leukocytes in the alveolar socket are summarized in Figure 2, Figure 3 and Figure 4. The number of rats decreased to 5–6 per group because we removed the data for those with remaining roots after tooth extraction. At 3 days after tooth extraction, in both groups, there was a small clot composed of red blood cells and fibrin and the clot was mainly replaced with granulation tissue. The experimental group showed higher collagen density (*p* < 0.05) and lower numbers of polymorphonuclear leukocytes (*p* < 0.05) in the upper part of the sockets, as compared to the control group. In the bottom and the middle parts of the sockets, there were no differences in collagen density or number of polymorphonuclear leukocytes between the experimental and the control groups at 3 days. At 8 days after tooth extraction, osteoid and woven bone were observed in both groups. No significant differences in collagen density and number of polymorphonuclear leukocytes in all parts of the socket between the control and the experimental groups were observed at 8 days.

**Figure 5:**
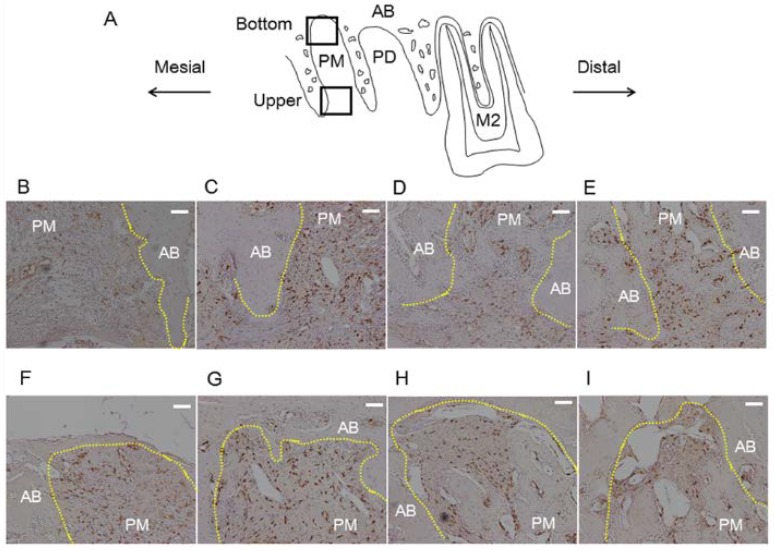
Schematic view and photographs of alveolar socket stained with FGF-2 (Scale bar = 100 μm). Schematic view of upper region and bottom region is shown in (**A**). Photographs of upper region are shown (**B**: 3-days control group; **C**: 3-days experimental group; **D**: 8-days control group; **E**: 8-days experimental group). Photographs of bottom region are shown (**F**: 3-days control group; **G**: 3-days experimental group; **H**: 8-days control group; **I**: 8-days experimental group). FGF-2-positive cells were stained brown. Expression of FGF-2 in the upper region, but not in the bottom region, was higher in the experimental group than in the control group at 3 days. On the other hand, there were no significant differences in FGF-2 expression in both regions between the groups at 8 days.

**Figure 6:**
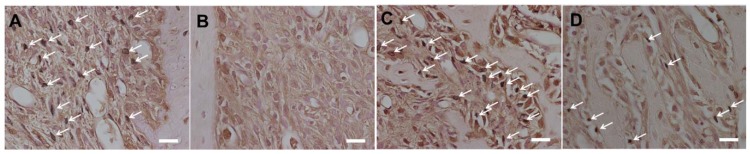
Photographs of alveolar socket stained with 8-OHdG (Scale bar = 20 μm). Photographs of upper region are shown (**A**: 3-days control group; **B**: 3-days experimental group; **C**: 8-days control group; **D**: 8-days experimental group). 8-OHdG-positive cells were stained brown (white arrows). Expression of 8-OHdG in the upper region was lower in the experimental group than in the control group at 3 and 8 days.

The experimental group also showed greater expression of FGF-2 in the upper part of the socket than in the control group at 3 days (Figure 5). However, there were no differences between the control and the experimental groups in FGF-2 expression in the bottom and the middle parts of the socket at 3 days and in all parts of the socket at 8 days. In addition, the experimental group showed lower expression of 8-OHdG in the socket than in the control group at 3 and 8 days (Figure 6).

### 3.3. Bone Morphogenetic Changes

**Figure 7:**
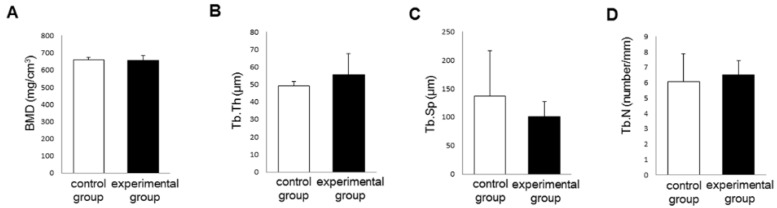
Bone morphogenetic analyses of left maxillae at 8 days. There were no significant differences in bone mineral density (**A**); trabecular thickness (**B**); trabecular separation (**C**) and trabecular number (**D**) between the experimental and control groups.

After removing the rats having remaining roots after tooth extraction, the number of rats decreased to 6 per group. There were no differences in bone mineral density, trabecular thickness, trabecular separation and trabecular number between the two groups at 8 days (Figure 7).

### 3.4. Gene Expression of Inflammation, Oxidative Stress and Collagen Turnover Markers

**Figure 8:**
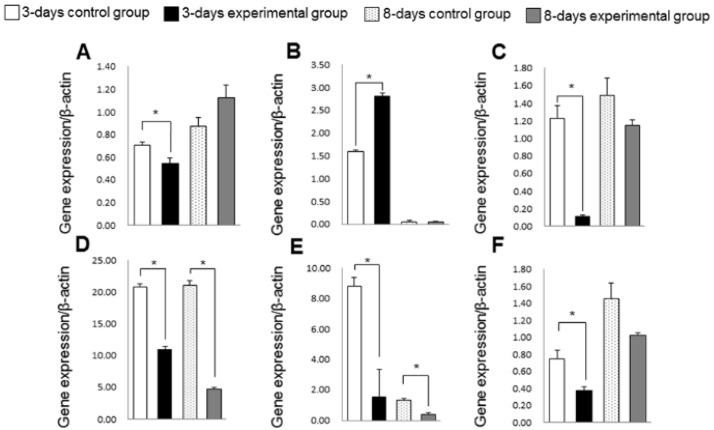
Gene expressions of matrix metalloproteinase-3 (MMP-3) (**A**); tissue inhibitor of metalloproteinase-1 (TIMP-1) (**B**); nuclear factor-κB (NF-κB) (**C**); interleukin-1β (IL-1β) (**D**); tumor necrosis factor-α (TNF-α) (**E**); heme oxygenase-1 (HO-1) (**F**) in rats. Bars represent means ± SD of 6 rats. * *p* < 0.05 compared with control group.

In the experimental group, gene expression of IL-1β, NF-κB, TNF-α and HO-1 was lower than in the control group at 3 days (*p* < 0.05) (Figure 8). The expression of these genes in the experimental group was apparently lower than in the control group at 8 days, although these differences in NF-κB and HO-1 expression were not statistically significant. The experimental group also showed higher expression of TIMP-1 and lower expression of MMP-3 than the control group at 3 days. However, there were no significant differences in TIMP-1 and MMP-3 expression between the groups at 8 days. 

## 4. Discussion

This is the first study to examine the effects of rCoQ10 on wound healing after tooth extraction. At 3 days after tooth extraction, the clot was mainly replaced with granulation tissue in both groups, and histological findings at 3 days were consistent with the granulation stage of wound healing [19]. The experimental group showed higher collagen density and lower numbers of polymorphonuclear leukocytes in the upper part of the socket when compared with the control group. Topical application of rCoQ10 may thus promote collagen production and suppress inflammatory reactions in the upper part of the socket during the granulation stage. On the other hand, there were no differences in collagen density and number of polymorphonuclear leukocytes at the bottom of the socket. In this study, ointment containing rCoQ10 was applied to the surface of the socket with a cotton ball. However, use of tools such as a syringe to inject rCoQ10 may be necessary to promote wound healing at the bottom of the socket.

Gene expression of HO-1, IL-1β, TNF-α and NF-κB, which are involved in oxidative stress and inflammation [20,21,22], was suppressed by rCoQ10 application at 3 days. In a pilot study, we also confirmed that total CoQ10 concentration in the periodontal tissue increased after topical application of ointment containing rCoQ10. Furthermore, protein expression of 8-OHdG was decreased by rCoQ10 application at 3 and 8 days. Based on these observations, it is conceivable that the anti-oxidative and anti-inflammatory effects of rCoQ10 directly contributed to wound healing in our model. It is known that exposure to oxidative stress impairs wound healing [3]. Topical application of rCoQ10 suppressed oxidative stress and its related inflammatory reactions, and this may accelerate wound healing. This is consistent with a previous study, which demonstrated that the anti-oxidative effects of CoQ10 induced cutaneous wound healing in mice [9]. In addition, it is also reported that CoQ10 can improve mitochondrial efficacy [7,8,23] and control inflammatory cytokines [24]. Such effects would also be beneficial to improve wound healing.

The experimental groups exhibited high expression of FGF-2 in the upper part of the socket at 3 days. Previous studies demonstrate that FGF-2 induces wound healing and tissue regeneration [25]. The increased production of FGF-2 following rCoQ10 application would also promote wound healing of the tooth extraction socket. In addition, the experimental group showed lower gene expression of MMP-3 and higher gene expression of TIMP-1, when compared with the control group. MMPs and TIMPs play an important role in extracellular matrix remodeling [26]. This suggests that rCoQ10 increases collagen density by controlling extracellular matrix remodeling. 

On the other hand, at 8 days after tooth extraction, osteoid and woven bone were observed in the socket. Thus, the histological findings at 8 days were indicative of the callus stage of wound healing. Therefore, healing of bone tissue is more important than that of soft tissue at 8 days. However, there were no bone morphogenetic differences between the experimental and control groups at 8 days. This indicates that topical application of rCoQ10 had little effect on bone remodeling in the socket. A previous study reported that CoQ10 suppresses osteoclast differentiation and enhances bone-forming osteoblast differentiation [27]. However, as collagen density is high at the callus stage, rCoQ10 may not penetrate deeply enough to stimulate bone remodeling in the socket. 

CoQ10 is a safe material with very low toxicity. Previous studies have shown that oral application of CoQ10 is effective at improving periodontitis [28]. Another study also found that rCoQ10 has anti-aging effects on periodontal tissues in healthy rats [13]. In the present study, we confirmed that topical application of rCoQ10 increased collagen density and decreased inflammatory reactions in the granulation stage during wound healing after tooth extraction. Although further studies are necessary, the use of oral rinse, dentifrice and ointment containing rCoQ10 may be beneficial for improving acute inflammation after tooth extraction. 

In this study, because rCoQ10 is fat-soluble, we used ointment containing soy bean oil. Previous studies have been shown that there is an inter-relationship between periodontal tissue and fat consumption [29,30,31,32,33]. These suggest that soy bean oil in the ointment may also affect the present results. Therefore, the effects of topical application of rCoQ10 may vary according to the type of vehicles.

Our study has some limitations. First, the study was only performed for 8 days. In rats, it takes about 1 month for the socket to heal completely. Therefore, extraction wound healing in rats is generally studied for more than 8 days [34,35,36]. However, we did not continue the study for a longer period, because there were no differences in wound healing between the experimental and control groups at 8 days. Second, we extracted maxillary first molars. Maxillary first molars have five roots and the mesial root has distal inclination. Therefore, it was difficult to extract maxillary first molars and some rats had remaining roots. In subsequent studies, teeth that are more easily extracted (e.g., maxillary second molars) should be selected. 

## 5. Conclusions

Topical application of rCoQ10 promotes wound healing of the soft tissue in the upper part of the alveolar socket, but the effects of rCoQ10 on bone remodeling were low in rats.
